# Contributions to Obstetric Jurisprudence

**Published:** 1859-01

**Authors:** Horatio R. Storer

**Affiliations:** Boston


ART. I.—*Contributions to Obstetric Jurisprudence.* By HORATIO R. STORER, M.D., of Boston.

NO. I.—CRIMINAL ABORTION.

*By the Common Law and by many of our State Codes, fœtal life, per se, is almost wholly ignored and its destruction unpunished;* abortion in every case being considered an offence mainly against the mother, and as such, unless fatal to her, a mere misdemeanor, or wholly disregarded.

*By the Moral Law,* THE WILFUL KILLING OF A HUMAN BEING AT ANY STAGE OF ITS EXISTENCE IS MURDER.

In undertaking the discussion at length of this subject, three preliminary facts must be assumed:—

*First.*—That if abortion be ever a crime, it is, of necessity, even in isolated cases, one of no small interest to moralist, jurist, and physician; and that when general and common, this interest is extended to the whole community and fearfully enhanced.

*Secondly.*—That if the latter assumption be true, both in premise and conclusion, neglected as the crime has been by most ethical writers and political economists, hastily passed over by medical jurists,* and confess-

---

* So far as the writer is aware, there exists, in this or any other language, no paper upon the subject at all commensurate with its importance. The chapters devoted to it in medical text-books, though some of them admirable so far as they go, especially that of Beck, are defective and often erroneous; while but little information of any value can be found elsewhere. In the French periodicals have appeared articles on special points hereafter referred to; in Great Britain able arguments regarding the commencement of fœtal life have been made by Radford, (1848;) and in this country, with remarks on the frequency of the crime, by Hodge, of Philaedly everywhere the great opprobrium of the law, often indeed by taunt that of medicine, either it cannot in the nature of things be suppressed, as by these facts implied, or its suppression has not been properly attempted. Discarding the former of these alternatives as alike unworthy of belief and proved false by facts hereafter to be shown, it will appear,

*Thirdly.*—That the discussion now broached is neither supererogatory nor out of place; further, that it is absolutely and necessarily demanded.

Moreover, in order that the importance and various bearings of the subject may be better appreciated, and that the writer's position and aims may be more fairly understood, it must be borne in mind that there exist to this discussion certain positive and apparent objections, which have, in a measure, given rise to much of the silence and omission alluded to above, and are, in the main, as follows:—

1. The natural dislike of any physician to enter upon a subject on some points of which it is probable that a portion of the profession is at variance with him, either from disbelief in the alleged increase of criminal abortion, unnoticed for reasons shown hereafter, or from a blind reliance on Providence of itself to abate the evil.

2. His fear, lest by any possible chance, by showing the frequency of the crime and its means, he may unhappily cause its still further increase.

3. The reluctance on the part of many of the profession to attack a powerful and acknowledged monied interest;

4. And to tell their patients, more commonly than is yet general, most unwelcome truth; thus not merely condemning, but, to their own consciences at least, criminating them;

5. And individually to risk losing practice, if thought more scrupulous than others;

6. And to be brought into more frequent contact with the law, even though for ends of justice;

7. And to exercise greater care and discretion in diagnosis and treatment, lest themselves be brought to answer for malpractice, or worse;

8. And publicly to discuss matters supposed to be generally unknown, and thus seem to throw open professional secrets to the world.

And, finally, grave doubts lest the statements made, though simple and true, should yet appear so astounding as to shock belief, or so degrading as to tend to lessen all faith in natural affection and general morality.

But these objections, so far at least as regards the profession, are un-

---

delphia, (1839 and 1854,) and by the present Professor of Obstetrics in Harvard University, (1855.) To the latter, his father, and to the journalists (Drs. Morland and Minot, of Boston,) by whom the effort then made was so warmly and eloquently seconded, the writer acknowledges his indebtedness for the thought of the present undertaking.

doubtedly but of limited existence; and, on the other hand, as more than counterbalancing them all, are the following arguments:—

That medical men are the physical guardians of women and their offspring; from their position and peculiar knowledge necessitated in all obstetric matters to regulate public sentiment and to govern the tribunals of justice.

That the discussion by them of this crime may very probably be the means, in great measure, of ultimately restraining or suppressing its perpetration.

That such will undoubtedly tend to save much health to the community and many human lives.

And, that, were there no other reason, it is clearly a duty.

I shall accordingly proceed to prove, so far as possible, the truth of every premise as yet stated, and to show the real nature and frequency of the crime: its causes; its victims; its perpetrators and its innocent abettors; its means and its proofs; its excuses, the deficiencies and errors of existing laws, and the various other obstacles to conviction; and, above all, so far as the present series of papers is concerned, the duty of the profession toward its general suppression.

### I. IS ABORTION EVER A CRIME?

That this could have been doubted, least of all by mothers, however ignorant or degraded, would at first sight appear improbable. The sense of the public, however, its practice, its laws, being each proved to the contrary by the stubborn evidence of facts, the necessity of our preliminary inquiry will be made manifest.

To postpone, for the present, all other considerations, we will regard abortion in the abstract. It may be defined, best perhaps, as the violent and premature expulsion of the product of conception, independently of its age, viability, and normal formation. These characteristics are eliminated as having judicially and actually nothing to do with the essential nature of abortion, whereas in infanticide they are each elements of great importance; a difference that will hereafter be seen.

We further, in the present investigation, set aside all cases where abortion is the result of accident, or from natural causes, or justified by the rules of medicine, whether to save the mother or her child. We shall have occasion, in the subsequent course of our inquiries, to discuss this latter question somewhat fully, and to set forth unpleasant truths. We now confine ourselves exclusively to those instances where the attempt at premature expulsion of the product of conception is artificially induced and intentional, and where, so far as can be judged, it is not necessitated and would not otherwise have occurred.

In the first place, the laws do not recognize that unnecessary abortion, *per se*, is a crime.

This act, when unnecessarily done, must be for one of two reasons: either to prevent the product of conception from receiving life, which subsequent evidence will show cannot be the case, or, if living, to destroy it.

We have said that the Common Law and many of our American statutes lose sight of this fundamental idea. Though based upon the first of the above alternatives,—the erroneous one, as regards the fact of their existence,—they are so worded as almost wholly to ignore fœtal life, to refuse it protection, to insure their own evasion, and by their inherent contradictions to extend the very crime they were framed to prevent.

They recognize, for the most part, no offence against the fœtus; we have just shown that such and such alone is always intended. They punish an attempt, which does not exist, upon the well-being or life of the mother; the intent being seldom or never to destroy the mother. She is herself, in almost every case, a party to the action performed; an accessory or the principal. To constitute a crime, a malicious or wicked intent is supposed to exist; we have thrown aside, as does the law, every case occurring from accident or from justifiable cause. The intent, if existing, as of course must be always the case, is against, and only against, the product of conception.

Again, the punishment meted by the law proves the truth of these propositions. Unless the woman die in consequence of the offence, it is declared, in every stage of pregnancy, a mere misdemeanor, as in Massachusetts; or else, while called such, or by omission justified or openly allowed in the early months when the fœtus is without other safeguard, the law pronounces abortion a felony and increases its penalties in more advanced pregnancy, after quickening has rendered it infinitely more certain that the fœtus will remain undisturbed, and has thus in the great majority of cases prevented the crime.

On the other hand, granting for the moment that the erroneous assumptions of the law were correct, and that the attempt were upon the life of the mother, how inconsistent to punish murder, attempted or committed, if by injury to the throat or heart, capitally, and if by injury to the womb, by temporary imprisonment; especially where this latter case always necessitates the slaughter of a second human victim.

Or, granting that the attempt were only upon the mother's health or temporary welfare, how absurd to punish the offence in early pregnancy, where her risks are greatest, by a trifling penalty or not at all, and in more advanced pregnancy, where these risks are daily lessened, with increased severity.

And, finally, if the fœtus were, as has been sometimes supposed, merely *pars viscerum matris*, its removal would be like that of a limb, or of any other portion of the body, whose loss is not absolutely attended with that of life; if made with the mother's consent, it would be unpunishable by law; if against her will, it would be already amenable, like other maim or mutilation, to existing statutes. In the one case, laws against abortion were needless; in the other, unjustifiable.

In a word, then, in the sight of the common law, and, in most cases of the statutory law, also, the crime of abortion, properly considered, does not exist; the law discussing and punishing a wholly supposititious offence, which not only does not exist, but the very idea of whose existence is simply absurd.

We turn now to public opinion. It, too, both in theory and in practice, fails to recognize the crime. Its practical denial of the true character of the offence will be shown in the course of our remarks on its frequency. Its theoretical denial we here consider, as proved in three ways—by implication, by collateral testimony, and by direct.

First, the maxims of the law are based on past or present public opinion. If merely on past, and this has totally changed, the law in matters of such importance is compelled to change also. The fact that the laws on this subject remain unaltered, if it be granted, as will be proved, that they are erroneous, furnishes us at the outset, and so far, with evidence that public opinion was formerly wrong, and that it so continues.

The frequency of the offence, and the character and standing of the mothers upon whose persons it is practised, accessories as we have seen, or principals, to it, furnish similar and more cogent testimony regarding the theory upon which it is founded. We shall soon perceive how extensive and high reaching is the frequency; we must therefore conclude that the public do not know, or knowing deny, the criminal character of the action performed.

Again, the direct testimony of the parties themselves is often available. It is undoubtedly a common experience, as has certainly been that of the writer, for a physician to be assured by his patients, often no doubt falsely, but frequently with sincerity, that their abortions have been induced in utter ignorance of the commission of wrong; in belief that the contents of the womb, so long as manifesting no perceptible sign of life, were but lifeless and inert matter; in other words, that being, previously to quickening, a mere ovarian excretion, they might be thrown off and expelled from the system as coolly and as guiltlessly as those from the bladder and rectum.

It having now been shown, directly and by temporary assumption, that the law and public sentiment, both by its theory and its practice, alike deny to unjustifiable abortion the imputation of crime, it remains for us to discuss this question abstractly, and to prove not merely that they are wrong, but that the offence is one of the deepest guilt, a crime SECOND TO NONE.

Ignorance of the law is held no excuse. The plea of ignorance of guilt could hardly better avail where its existence is implied by common sense, by analogy, and by all natural instinct, binding even on brutes. Were this guilt, however, clearly shown, and its knowledge, supposed wanting, to be spread broadcast by the press, the all-powerful arbiter of public opinion, the last and strongest prop of the crime were gone.

It has been shown, by setting aside all accidental cases, those naturally occurring and those necessarily, and in the absence of reasonable evidence to the contrary, that all other abortions must be intentional, that they must be occasioned by the "malice aforethought" of the law. It has also been shown that in these cases, except it exist as an additional element, the malicious intention is not against the life or person of the mother, but that in every instance it is against the product of her womb. Hence, the whole question of the criminality of the offence turns on this one fact, the real nature of the fœtus in utero. If the fœtus be a lifeless excretion, however soon it might have received life, the offence is comparatively as *nothing;* if the fœtus be already, and from the very outset, a human being, alive, however early its stage of development, and existing independently of its mother, though drawing its sustenance from her, the offence becomes, in every stage of pregnancy, MURDER.

"Every act of procuring abortion," rules Judge King, of Philadelphia,* contrary to the usual interpretation of the law, "is murder, whether the person perpetrating such act intended to kill the woman, or merely feloniously to destroy the fruit of her womb."

Common sense, we have said, would lead us to the conclusion that the fœtus is from the very outset a living and distinct being. It is alike absurd to suppose identity of bodies and independence of life, or independence of bodies and identity of life; the mother and the child within her, in abstract existence, must be entirely identical from conception to birth, or entirely distinct. Allowing, then, as must be done, that the ovum does not originate in the uterus; that for a time, however slight, during its passage through the Fallopian tube, its connection with the mother is wholly broken; that its subsequent history is one merely of development, its attachment merely for nutrition and shelter, it is not rational to suppose that its total independence, thus once established, be-

* As quoted by Hodge. Introductory Lecture, p. 15.

comes again merged into total identity, however temporary; or that life, depending not on nine months' growth, nor on birth, because confessedly existing long before the latter period,—since quickening at least, a time varying within wide limits,—dates from any other epoch than conception; while it is as irrational to think that the influence of the father, mental and moral as well as physical, so often and so plainly manifested, can be exercised by any possibility upon the child at any other moment than that original and only one of impregnation itself.

Another argument is furnished us, similar, but differing. The fœtus, previous to quickening, as after it, must exist in one of two states, either death or life. The former cannot take place, nor can it ever exist, except as a finality. If its signs do not at once manifest themselves, as is generally the case, and the fœtus be retained in utero, it must either become mummified or disintegrated; it can never again become vivified. If, therefore, death has not taken place, and we can conceive no other state of the fœtus save one, that, namely life, must exist from the beginning.

These reasonings are strengthened by the evidence of analogy. The utter loss of direct influence by the female bird upon its offspring from the time the egg has left her, and the marked effect, originally, of the male; the independence in body, in movement, and in life, of young marsupial mammals, almost from the very moment of their conception, identical analogically with the intra-uterine state of other embryos,—nourishment by teat merely replacing that by placenta at an earlier period; the same in birds, shown by movements in their egg on cold immersion before the end of incubation; the permanence of low vitality, or of impaired or distorted nervous force, arising from early arrest or error of development, and necessarily contemporaneous with it, are all instances in point.

Brute instincts are often thought wholly supplanted by human reason. That this is not so is proved by what obtains in the absence of reason, whether from the outset or subsequently occurring. Idiots and lunatics alike show the actual identity in this respect of man and the brute, however instinct, in the former, may normally be tempered by conscience and reason. Whatever ideas on the subject of abortion the human mind may have forced itself to entertain, let the slightest proof concerning the existence of fœtal life be alleged, and maternal instinct at once makes itself known: the parent, as after its birth, would often even perish to preserve her child. This is not conscience, which is stirred only by an afterthought, but instinct.

Thus far, incidental proof concerning the commencement of fœtal life, and so the guilt of unjustifiable abortion. More decisive evidence is at hand.

That the movements of the fœtus, subsequent to quickening, whatever the actual nature of that first sensation may be, declare the existence of intra-uterine life, is allowed by the world; by none more than by mothers themselves, whose statistics prove that after the perception of these movements criminal abortions are comparatively rare.

But quickening,—a period usually occurring from the one hundred and fifteenth to the one hundred and thirtieth day after conception, but varying within still more appreciable limits in different women, and in the same women in different pregnancies, from variations in the amount of liquor amnii, the early strength of the fœtus, and other causes, and also, if at all, owing in its first sensation to rising of the womb from the pelvis, probably occurring a little earlier with boys than with girls, from their relative difference in size,*—is often absent, even throughout pregnancy; and fœtal movements are sometimes appreciable to the attendant when not to the mother, or indeed to the mother alone.

Further, in premature births, where quickening has not occurred, or before its usual period, by the movements of the fœtus, its earlier independent and vital existence is sometimes reduced to a matter of ocular demonstration; while to the ear, in very many instances, as early and as conclusive evidence is afforded by the sounds these movements give rise to.†

Quickening is therefore as unlikely a period for the commencement of fœtal life as those others set by Hippocrates and his successors, varying from the third day after conception, to that of the Stoics, namely birth, and as false as them all.

We need not, with Dubois‡ and some earlier writers,§ from the manifest relation of means to the end, consider that the movements of the fœtus in utero, and its consequent attitude and position, are signs of an already developed and decided sentience and will, nor is it requisite to suppose them the effect of an almost rational instinct. But that they are wholly independent of the will and the consciousness of the mother, and yet by no means characteristic of organic life, whether hers or its own,—which latter is also by abundant evidence proved independently to exist,—but decidedly

---

* Hippocrates states that this is a fact, and that he had found the difference of a whole month, which he attributes to the "greater strength" of the male.—(*On the Nature of the Child*, Sect. 11.) I am unaware that this point has been investigated by any modern writer

† "These sounds may sometimes be distinguished *several weeks* before the mother becomes conscious of the motions of the child."—NAEGELE; *Treatise on Obstetric Auscultation*, p. 50.

‡ Memoire sur la cause des Présentations de la Tête, &c.—*Mém. de l'Acad. Roy. de Méd.* tome ii.

§ A. PARÉ; English Trans., p. 899. HUGH CHAMBERLEN'S Trans. of Mauriceau on *Diseases of Women with Child*, p. 147, Note. ENNEMOSER; Historisch-physiologische Untersuchungen über den Ursprung und das Wesen der menschlichen Seele; Bonn, 1824. CABANIS; Rapports du Physique et du moral de l'Homme, tome ii. p. 431.

animal in their character; that they are not explainable by gravity, despite all the arguments alleged, latest by Matthews Duncan,* nor on any other supposition save that of a special and independent excito-motory system, distinct from that of the mother,† brings us directly down to this—the existence of as distinct and independent a nervous centre, self-existing, self-acting, living.

We set aside all the speculations of metaphysicians regarding moral accountability of the fœtus, the "potential man," and its "inanimate vitalities," as useless as they are bewildering. If there be life, then also the existence, however undeveloped, of an intellectual, moral, and spiritual nature, the inalienable attribute of humanity, is implied.

If we have proved the existence of fœtal life before quickening has taken place or can take place, and by all analogy and a close and conclusive process of induction, its commencement at the very beginning, at conception itself, we are compelled to believe unjustifiable abortion always a crime.

And now words fail. Of the mother, by consent or by her own hand, imbrued with her infant's blood; of the equally guilty father, who counsels or allows the crime; of the wretches who by their wholesale murders far out-Herod Burke and Hare; of the public sentiment which palliates, pardons, and would even praise this so common violation of all law, human and divine, of all instinct, of all reason, all pity, all mercy, all love, we leave those to speak who can.

We have next to prove the frequency of abortion.

[TO BE CONTINUED.]

The writer, having been directed by the American Medical Association, at its meeting at Nashville in 1857, to prepare a Report on Criminal Abortion, with a view to its general suppression, from which duty he has hitherto been prevented by ill-health, desires the general coöperation of the profession. He has already received pertinent and valuable information from many parts of the Union, and would gladly hear from all who may be interested in the subject. For this purpose he subjoins the following inquiries:—

1. Is Criminal Abortion, whether induced by the patient herself or not, on the increase in your neighborhood?

2. Is it confined to unmarried women?

3. If it is increasing, is further silence and inaction the duty of the profession?

4. Is a general exposure of its true character, owing as it often is to ignorance, and a decided and general denouncement of its guilt, likely to increase still further the crime?

5. Is it necessary and advisable, where the laws on this subject are notoriously and fundamentally defective, for the profession to recommend their revisal and subsequent enforcement?

---

* Ed. *Med. and Surg. Journ.*, Jan. 1855, p. 50.

† SIMPSON; *Obstetric Works*, vol. ii. p. 88.

